# miR-93-5p suppresses ovarian cancer malignancy and negatively regulate CCND2 by binding to its 3′UTR region

**DOI:** 10.1007/s12672-022-00478-1

**Published:** 2022-03-20

**Authors:** Guotong Chen, Yiwei Yan, Xiaojv Qiu, Chengfeng Ye, Xingmei Jiang, Shuo Song, Yibo Zhang, Huanhuan Chang, Leqi Wang, Xuehuan He, Lingrong Tang, Qingyu Zhang, Ying Zhang

**Affiliations:** 1 Department of Obstetrics and Gynecology, Affiliated Hospital of Guangdong Medical University, Zhanjiang, China; 2 Graduate School of Guangdong Medical University, Zhanjiang, China; 3 The Marine Biomedical Research Institute, Guangdong Medical University, Zhanjiang, China; 4 Department of Cell Biology, College of Life Science and Technology, Jinan University, Guangzhou, China

## Abstract

Ovarian cancer is the most fatal gynecological cancer worldwide, yet the fundamental mechanism of malignancy acquisition in ovarian cancer remains unknown. miRNA has been implicated to a variety of diseases, including cancer initiation and progression. Cyclin-D2 (CCND2) is ubiquitously implicated in cancer uncontrol cell proliferation. Bioinformatic research revealed that CCND2 is a candidate gene for miR-93-5p with a binding site in its 3′UTR region in the current study. Using our ovarian cancer sample, we verified that miR-93-5p is negatively correlated with CCND2 mRNA and protein levels. Luciferase report assay revealed miR-93-5p inhibits CCND2 production through binding to the 3′UTR region. The expression of miR-93-5p in ovarian cancer patient samples was then determined, and a survival analysis was performed. Our findings showed that miR-93-5p is downregulated in ovarian cancer and is a favorable predictive factor in ovarian cancer patient. CCK8 assay, wound healing assay and flow cytometry-based cell cycle and apoptotic cell analyses were employed here. We found that miR-93-5p suppresses ovarian cancer cell proliferation and migration while enhances cell death. Our research certified that miR-93-5p reduces ovarian cancer malignancy by targeting CCND2.

## Introduction

Ovarian cancer is the most lethal gynecological disease. It is estimated that there are about 240,000 new cases worldwide every year, which account for about 2.5% of total cancer cases but lead to over 150,000 cases of death toll every year [1]. The early symptom of ovarian cancer is not significant and always miss diagnosed with intestinal disease. Early screen by transvaginal ultrasound (TVUS) and the CA-125 blood test but the TVUS only for a big mass of tumor while CA125 specificity is low [2]. Therefore, 75% of patients are diagnosed at an advanced stage, and chemotherapy if not the only choice at least is an irreplaceable intervention option [3, 4].

miRNA is a class of single-stranded non-coding ribonucleotides with a length of approximately 22 nucleotides, which is always transcript by a complete endogenous gene and functions as gene expression regulators of parental genes [5]. It is predicted over 60% of human genes are regulated by miRNA through binding to the 3'untranslated region (3′UTR) or coding region of the target gene mRNA [6]. MicroRNA can simultaneously regulate multiple target genes which leads to cell adaptive change to the microenvironment. It has been reported that microRNA is implicated in cancer progression in various of cancer for example miR-135a-3p, miR-200c, miR-216a, and miR-340 [7, 8]. miRNA could be promising target for cancer early diagnosis and prognosis.

CCND2 is a member of the cyclin family and is a key positive regulator of the mammalian cell cycle. CCND2 is ubiquitously involved in cancer cell proliferation [9]. The complex formed by CCND2 and CDK4/6 (CCND2–CDK4/6) regulates the phosphorylation and inactivation of tumor suppressor retinoblastoma protein (RB) to prompt cells transition from G1 phase to S phase [10]. CCND2 upregulated and could be a therapeutic target for a variety of tumors, including colon cancer, stomach cancer, liver cancer, breast cancer and lung cancer as well as ovarian cancer. Although CCND2 function are well-known, the mechanism of CCND2 expression, degradation in ovarian cancer remains unclear.

In this study, we found that the expression of miR-93-5p in cancer tissues is lower than that in normal tissues. The target binding prediction suggests that *CCND2* may be the target gene of miR-93-5p. Transfection miR-93-5p mimics to ovarian cancer cells can downregulate the *CCND2* gene expression and its protein levels. We confirmed miR-93-5p is a negative regulator for malignancy in term of cell proliferation and cell survival. The data from the clinical sample suggested miRNA -93-5p has a negative correlation with CCND2 levels in cancer patients. Our results identified that miR-93-5p inhibit ovarian cancer malignancy by regulating CCND2.

## Materials and methods

### Patient and tissue samples

Human ovarian cancer and normal ovarian tissues samples were obtained from the Affiliated Hospital of Guangdong Medical University (Guangdong, China) from January 2019 to June 2020. This research was approved and supervised by the Hospital Ethics Committee of the Affiliated Hospital of Guangdong Medical University. All patients have signed the informed consent to agree to the use of tissue samples for research purposes. All patients had not received radiotherapy or chemotherapy before surgery, and the pathology diagnosis of the samples was determined according to postoperative tissue immunohistochemistry.

### Cell culture

Human epithelial ovarian cancer cell line A2780 was obtained from the Shanghai Gaining Biological Ltd. This cell line was cultured in RPMI-1640 supplemented with 10% fetal bovine serum (FBS). Human normal ovarian epithelial cell line IOSE80 was obtained from the BeNa Culture Collection Ltd. This cell line was cultured in DMEM supplemented with 10% fetal bovine serum (FBS). HM cell is a high metastatic ovarian cancer cell line which was kindly gifted from Professor Alice Wong of Hongkong University. HM cells was maintained in DMEM supplemented with 10% FBS. All the cell lines were maintained at 37 °C in a 5% CO_2_ humidified atmosphere.

### RNA extraction, cDNA synthesis and qPCR

Total RNA was extracted from tissues or cells using Trizol (Takara, Japan) and cDNA prepared and subsequently quantitated using Mir-X™ miRNA First-Strand Synthesis and TB Green^®^ qRT-PCR (Takara, Japan) performed on ABI 7500 qRT-PCR System U6 was used as the internal control for quantitative PCR of miRNA, while the beta-actin was used as internal control used for quantitative PCR of CCND2. 2-delta delta CT was used to calculate the gene level in each sample. The primers used in this study were listed below (Table 1).

**Table 1:** Primer sequences used for qPCR

Primer name	Sequences (5′–3′)
miR-93-5p	F: GCCGCCAAAGTGCTGTTC
	R: CAGAGC AGGGTCCGAGGTA
U6	F: CTCGCTTCGGCAGCACA
	R: AACGCTTCACGAATTTGCGT
CCND2	F: TTGTGATGCCCTGACTGAGC
	R: CACGTTGGTCCTGACGGTACT
ACTIN	F: AGCGAGCATCCCCCAAAGTT
	R: GGGCACGAAGGCTCATCATT

### Western blot

Cells or tissues proteins were extracted using precooled RIPA lysis buffer. Sample lysed and centrifugate for 5 min at 12,000 rpm, 4 ℃. Collect the protein supernatant and then add loading buffer to prepare the protein sample for the WB experiment. Proteins were separated by 10% SDS-PAGE electrophoresis and transferred to polyvinylidene difluoride (PVDF) membranes. Membranes were blocked with 5% no-fat milk for 1 h, then cut the required protein bands (CCND2 31 kDa, α-Tubulin 52 kDa) and incubated with primary antibodies (1:1000, CST) at 4 °C overnight. Membranes then were washed with TBST and incubated for 1 h with secondary antibodies (1:3000, CST) at room temperature. Enhanced chemiluminescence (ECL) kit and Canon 5200 were used to visualize the protein signal.

### Construction and Transfection of miRNA

The miR-NC and miR-93-5p mimic were purchased from Shanghai GenePharma Co., Ltd. (Shanghai, China). miRNA were transfected with Lipofectamine 3000 (Invitrogen, Carlsbad, CA, USA) when the density is about 50%.

### CCK8 assay

A total of 5000 cells were pre-seeded in each well of the 96-well plates. The cells were transfected with miRNA when cells confluence up to 80%. After 24 h, 48 h, and 72 h transfection, 10 μl of CCK8 solution (Yeasen, China) was added to each well and incubated for 1 h and read the absorbance at 450 nm by a microplate reader.

### Cell cycle analysis

To analyze the cell cycle distribution, cells were plated in 12-well plates at a density of 10 × 10^4^ cells per well, transfected after the cells were adhered to the wall. 48 h after transfection, the cells were digested with trypsin. Washed and centrifuged with precooled PBS then discarded the supernatant. 1 ml 70% ethanol was added into the centrifuge tube to resuspend cells and fix the cell at 4 ℃ for 24 h. After fixation, cells were washed with PBS and centrifuged, discarded the supernatant, and PI (Beyotime Biotechnology, China) staining for 30 min and then detect by flow cytometry.

### Wound healing assay

A total of 5 × 10^5^ cells were seeded in each well of the 6-well plates, transfected after the cells were adhered to the wall. After 24 h of transfection, cleaned the cell culture plate twice with PBS, then scratch from top to bottom with the end of a sterile pipette tip. Rinsed gently with PBS twice and then added an appropriate amount of 2% fetal bovine serum culture medium. Took pictures of cell scratches in 0 and 48 h, then analyzed cell scratch healing rate by Image J.

### Apoptosis analysis

The cells were seeded in 12-well plates at a density of 10 × 10^4^ cells per well overnight and cells were transfected with miRNA. After 48 h of transfection, the cells were digested with trypsin and collected. Cells Washed and centrifuged with precooled PBS then discarded the supernatant 2 times. Annexin V FITC Apoptosis kit (BD, USA) was used for apoptotic cell analysis. The cells were resuspended in 500 μl staining buffer and stained with 10 μl of each Annexin V FITC and PI for 15 min protecting from light at room temperature. The well-stained cells were analyzed by flow cytometry.

### Luciferase report assay

To verify the target between CCND2 and miR-93-5p, the CCND2 wild(CCND2-wt) and mutant CCND2 (CCND2-mut) vectors were synthesized by CCND2 cDNA fragment insertion which contains the wild or mutant binding site of miR-93-5p into the pmirGLO dual-luciferase miRNA target expression vectors. Furthermore, 293 T cells were co-transfected with pmirGLO dual-luciferase reporter vectors (CCND2-wt and CCND2-mut) and miR-93-5p mimics or negative control. After 48 h, cells were harvested, and the luciferase activity was measured using the dual-luciferase assay system (Beyotime Biotechnology, China) following the instruction of manufacture, relative to that renilla luciferase.

### Immunohistochemistry

The collected specimens were fixed in 4% paraformaldehyde for at least 72 h and then dehydrated within an automatic dehydrator. Samples were embedded in liquid paraffin and store at 4 °C overnight. The tissues were then sliced 3 μm thick. The specimens were heated at 60 °C for 2 h and then deparaffinized in xylene and rehydrated in a series of graded alcohol. The sample was pretreated in 0.01 M Citrate buffer and make a heat-induced antigen retrieval was carried out by using the microwave to heat buffer to boil for 15 min. The slices were then incubated in 3% H_2_O_2_ and 0.1% Tween 20 at room temperature without light for 20 min to block endogenous peroxidase activity. Then slices were blocked by 5% goat serum for 30 min. Afterward, a primary antibody was incubated at 4 °C overnight. Subsequently, they were incubated with a secondary antibody for 1 h. 3,3ʹ-diaminobenzidine (DAB) chromogen was applied and then stained with hematoxylin. The slices then were dehydrated through graded alcohol and cleared in xylene and photographed under a microscope. The investigator conducting IHC (Xiaojv Qiu, Chengfeng Ye) were blinded to patient clinical data. The grading criteria were as follows: negative 0, weak 1, moderate 2, and strong 3. IHC scores were calculated as the mean by two pathologists.

### Statistical analysis

The result was expressed as mean ± standard error. Test the normality and variance equivalence of all data to determine the appropriate statistical test. A nonparametric test or independent sample T-test was used to determine significant differences in the group. The data were analyzed using Prism 8 (GraphPad, La Jolla, CA, USA). P < 0.05 was considered statistical significance.

## Results

### miR-93-5p downregulated in ovarian cancer cell and negatively regulates cell cycle booster CCND2

**Fig. 1:**
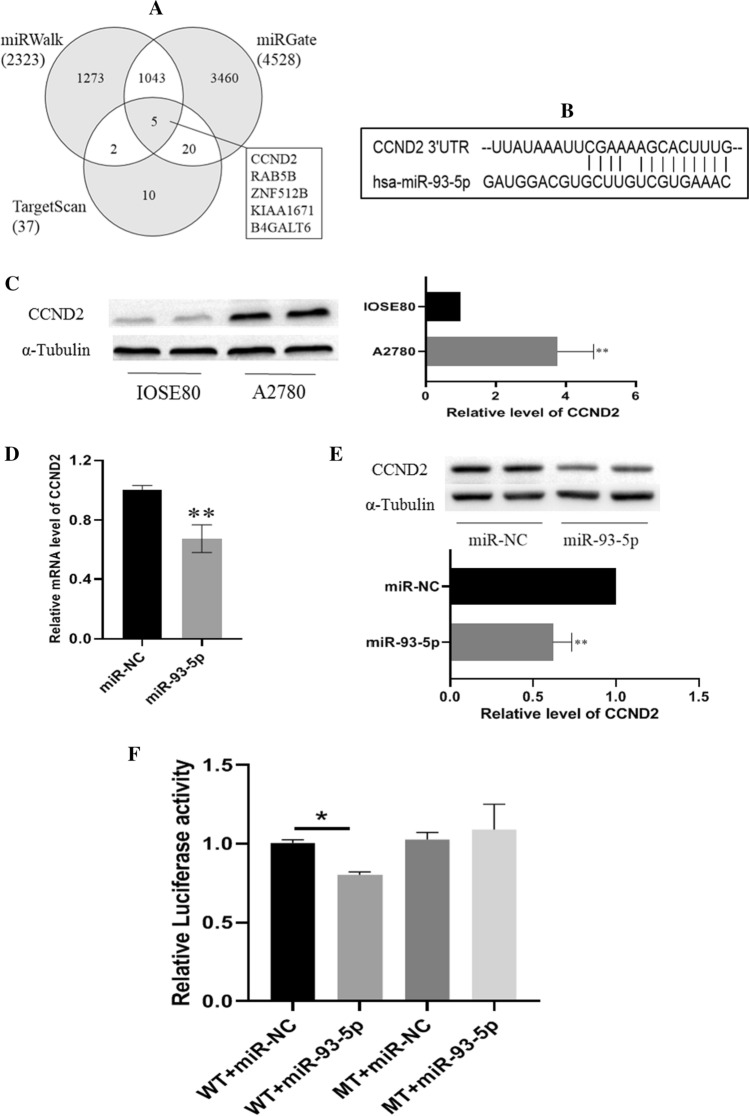
miR-93-5p downregulatesCCND2 by binding 3′UTR of CCND2 mRNA. **A** TargetScan, miRGate, miRWalk were used to screen target gene of miR-93-5p. **B** Prediction of target binding sites between miR-93-5p and CCND2 by using database RNA22v2. **C** CCND2 protein level in ovarian cancer cell A2780 and ovary immortalized cell IOSE80 was measured by western blot. Tubulin was used as internal control. **D** The qPCR assay was performed to detect the mRNA expression of CCND2 after miR-93-5p mimic or miR-NC transfection. Tubulin were used as internal control. **P < 0.01. **E** Western Blot was used to detect the CCND2 protein level after transfection of miR-93-5p mimic or miR-NC, **P < 0.01. **F** The luciferase activity that had been fused with wild type or mutant 3′UTR of CCND2 were tested by luciferase reporter assay, *P < 0.05

To answer why miR-93-5p suppresses ovarian cancer cell proliferation and cell cycle arrest, we screened the potential target of miR-93-5p in three miRNA public databases including TargetScan, miRGate, miRWalk. We found out 5 potential targets in the three databases by Venn plot analysis. The 5 candidates are listed on the right of Fig. 1A and CCND2 is a well-known oncogenic gene involved in cell cycle G1/S transition. We predicated the binding site of miR-93-5p using the RNA22v2 tool (https://cm.jefferson.edu/rna22/Interactive/) and the result indicated miR-93-5p can bind the 3’UTR of CCND2 mRNA (Fig. 1B). We detected CCND2 in ovarian cancer cell A2780 and immortal ovarian epithelial cell IOSE8 and we found CCND2 in A2780 cell is higher than in IOSE8 (Fig. 1C). Thereby, we quantified the miR-93-5p level in both cell lines by qPCR. The qPCR result suggested the miR-93-5p level in A2780 is lower than the IOSE8 cell line (Fig. 1D). Transfection of miR-93-5p mimics into A2780 cells can significantly reduce the CCND2 protein level (Fig. 1E). To confirm whether miR-93-5p can regulate CCND2, CCND2 3′UTR was constructed into pmirGLO Dual-Luciferase miRNA Target Expression Vector. A mutant of 3′UTR was also constructed into the vector as a control. After transfection of miR-93-5p mimic or miR-NC, the luciferase activity was detected, and results show miR-93-5p only can significantly reduce firefly luciferase in the 3′UTR of WT CCND2 mRNA (Fig. 1F).

### miR-93-5p suppresses ovarian cancer cell proliferation

**Fig. 2:**
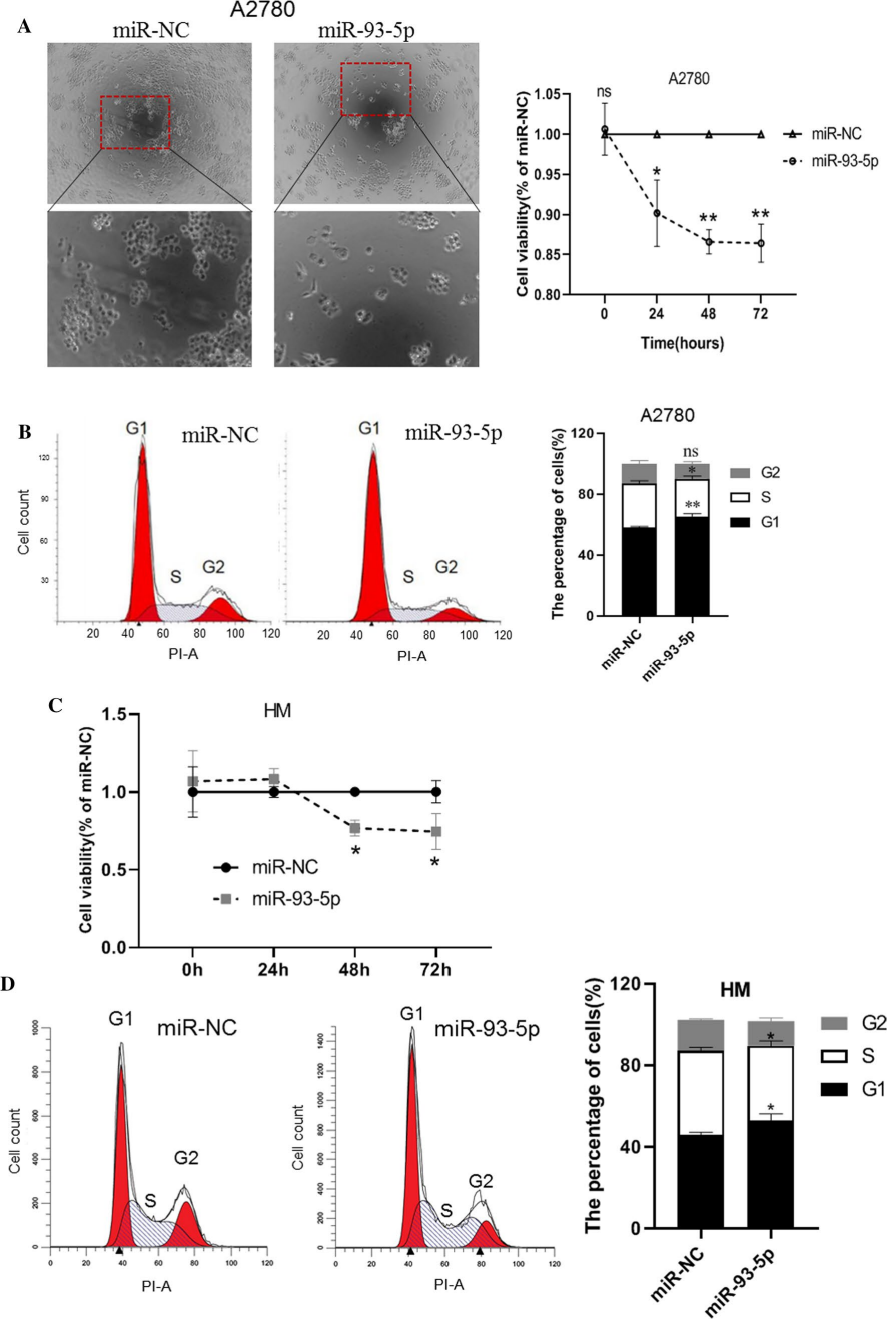
miR-93-5p suppresses ovarian cancer cell proliferation. **A** The morphology of A2780 cells under phrase contrast microscope after treatment of either miR-NC or miR-93-5p mimic for 48 h (left) andCCK8 assay was performed to measure cell viability after treatment of miR-93-5p mimic and miR-NC in A2780 cells (right). **B.** Cell cycle analysis was performed using PI staining and flow cytometry in A2780 cells. **C** CCK8 assay was performed to measure cell viability after treatment of miR-93-5p mimic and miR-NC in HM cells **D** Cell cycle analysis was performed using PI staining and flow cytometry in HM cells. *P < 0.05; **P < 0.01

To investigate the function of miR-93-5p in ovarian cancer cell malignancy, we transfection of miR-93-5p mimics to A2780 cell. After miR-93-5p transfection for 24 h, cell morphology was observed under a phase-contrast microscope (Fig. 2A) and a CCK8 assay was performed to verify the cell proliferation. The result showed miR-93-5p mimic transfection can significantly reduce cell viability in a time-dependent manner (48 h, 72 h p < 0.01, Fig. 2A right). Therefore, we wonder if miR-93-5p caused cell proliferation reduction result from cell cycle arrest. We test the cell cycle in the miR-93-5p and miR-NC group and the result showed miR-93-5p transfection increases G1 phase cell and reduces S phase cell, which suggests that miR-93-5p could inhibit cell proliferation by blocking the cell cycle at G1/S transition (Fig. 2B). To confirm the miR-93-5p function in cell proliferation, we used high metastatic ovarian cancer cell line HM cell to verify. Result showed that transfection of miR-93-5p into HM cell can significantly suppress cell proliferation (Fig. 2C) and lead to cell G1/S transition arrest compared to miR-NC transfection (Fig. 2D).

### miRNA-93-5p weakly increase ovarian cancer cell apoptosis and suppress cell migration

**Fig. 3:**
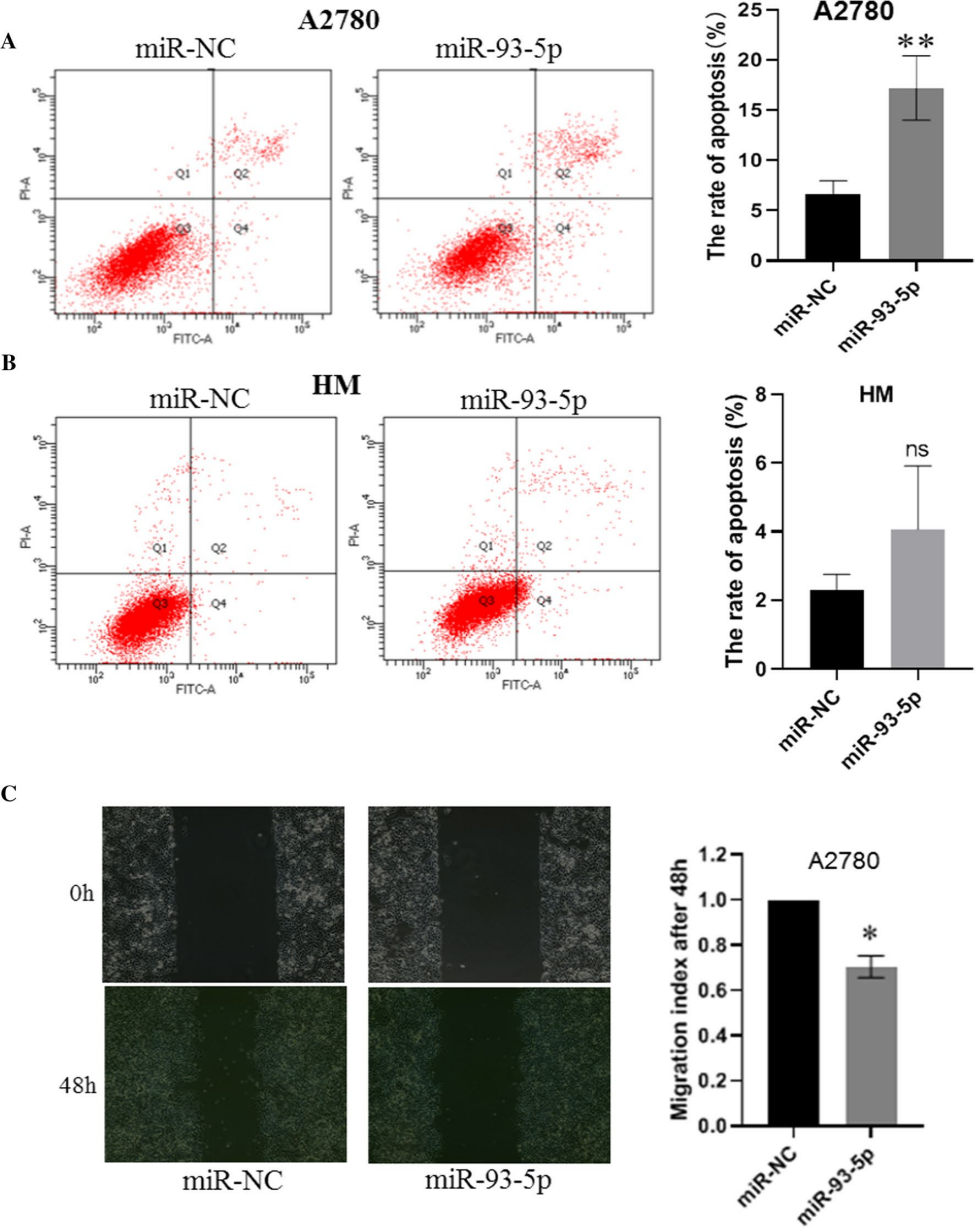
miRNA-93-5p enhances ovarian cancer cell apoptosis and suppress cell migration. **A**, **B** Cell apoptosis was analyzed by using AnnexinV-FITC/PI kit based on flow cytometry approach after cell transfection of miR-93-5p mimic and miR-NC in A2780 cells and HM cells. **C** Wound healing assay were performed to test the cell migration ability after transfection of miR-93-5p mimic and miR-NC in A2780 cell. *P < 0.05; **P < 0.01

We observed transfection of miR-93-5p induces cell death and hence we wonder whether miR-93-5p also can induce cell apoptosis. Cell apoptosis analysis indicated that miR-93-5p increases cell apoptosis up to 3-folds compared to the miR-NC group in A2780 cells (Fig. 3A). But miR-93-5p only increase about 50% cell death in HM cells which may cause of HM cell is high metastatic ovarian cancer cell with higher malignancy (Fig. 3B). We further test miR-93-5p function on cell migration, wound healing assay was performed, and the result showed miR-93-5p also inhibits A2780 cell migration but is not that significant as the cell proliferation and cell apoptosis (Fig. 3C).

### miRNA-93-5p is a positive prognosis marker and was downregulated in ovarian cancer

**Fig. 4:**
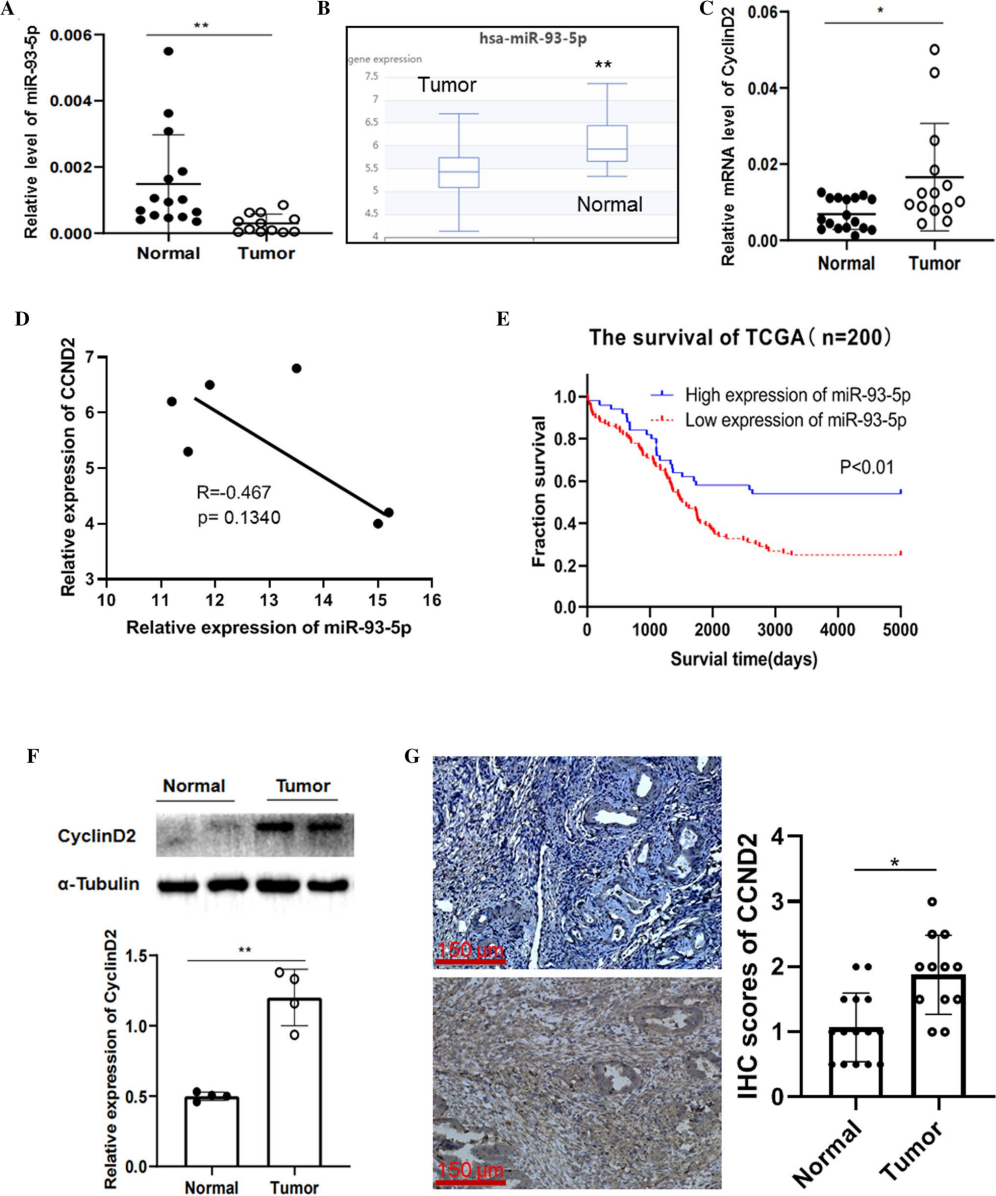
miR-93-5p is downregulated in ovarian cancer and predicate good outcome of patients. **A** The qPCR was applied to measure the expression of miR-93-5p in ovarian cancer tissues and normal ovary tissues (**P < 0.01). **B** The expression of miR-93-5p in ovarian tumor and normal health individual were extracted from the dbDEMC database (Experiment ID, EXP00625, **P < 0.01). **C** The qPCR was employed to measure the expression of CCND2 in ovarian cancer tissues and normal ovary tissues (*P < 0.05). **D** The Pearson analysis was performed to mRNA of CCND2 and miR-93-5p in tumor tissues. **E** The survival data and miR93-5p data were extracted from TCGA database and Kaplan–Meier Plotter analysis was performed (**P < 0.01). **F** Western blot were carried out to measure the CCND2 protein level in ovarian tumor tissues and normal ovary tissues (**P < 0.01). **G** IHC experiment was applied to detect the CCND2 in ovarian cancer tissue and normal ovary tissues, Scale bar = 150 μm (*P < 0.05)

We measured the miR-93-5p level in ovarian cancer tumor tissues using qPCR assay. Specific primer was used for qPCR and the melt curve was confirmed the primer is specific to the miR-93-5p. The result showed the miR-93-5p level in tumor cells is dramatically decreased (Fig. 4A). Bioinformatic analysis showed miR-93-5p was downregulated in ovarian cancer tissues compared to normal ovary (Fig. 4B). Meanwhile, we found that CCND2 was significantly increased in ovarian cancer tumor tissues accordingly (Fig. 4C). To investigate the relationship between the miR-93-5p and CCND2 in tumor tissues, correlation analysis was performed and the result suggest that miRNA-93-5p is negatively correlated with mRNA level of CCND2 (n = 6, R = − 0.467, p = 0.134, Fig. 4D). To explore the role of miR-93-5p in ovarian cancer progression, we utilized the data from the project of The Cancer Genome Atlas and make the survival curve of patients with the different expression levels of miR-93-5p. The result clearly shows that low expression of miR-93-5p is a poor prognosis factor of patient survival time (Fig. 4E, n = 200, p < 0.01). To confirm the pattern of CCND2 in tumor tissues, we further analyzed CCND2 levels in tumor tissues by both western blot and IHC assay. Immunoblot result suggests CCND2 upregulated in tumor (n = 4) compared to normal ovary tissues (n = 4) (Fig. 4F). IHC result clear also shows CCND2 level in tumor tissues (n = 12) are higher than non-tumor ovary tissues (n = 15) (Fig. 4G).

## Discussion

Since the first miRNA was discovered in 1993, the understanding of miRNA biology in development and disease has greatly increased, especially in many cancer cases, which affect the cell cycle, differentiation, apoptosis, migration and drug resistance so on and so forth [11]. As a regulatory molecule for homologous genes, it is predicted that more than 60% of human protein-coding gene mRNA dynamic level is regulated by miRNA [6]. Although miRNA is small but more stable than mRNA in blood, urine and other body fluids. The detection of miRNA levels in corresponding body fluids has the potential for early cancer diagnosis and predicting the prognosis of treatment. miRNA could serve as a novel of biomarker for cancer diagnosis and prognosis [12, 13]. Our research found that miR-93-5p is low expressed in ovarian cancer and is related to prognosis. However, due to its low expression, combining with tumor-related indicators such as CA125, CA199 would help to improve clinical diagnosis and prognosis of ovarian cancer.

We found that the expression of miR-93-5p is significantly down-regulated in ovarian cancer and is a protective factor for ovarian cancer patients. Functional experiments indicated miR-93-5p can significantly inhibit the malignant characteristics of ovarian cancer. It is reported that miR-93-5p has a differential pattern in a variety of malignant tumors, especially in digestive system tumors. The low expression of miR-93 in colon cancer is closely related to its metastasis, differentiation and poor prognosis. Up-regulation of miR-93 expression can inhibit the metastasis and invasion of colon cancer by inhibiting the Wnt/β-catenin signaling pathway [14]. In liver cancer, miR-93 can negatively regulate the expression of the tumor suppressor gene PTEN, and promote the proliferation, invasion and migration of hepatocellular carcinoma cells by activating the c-Met/PI3K/Akt signaling pathway [15]. In gastric cancer, miR-93 negatively regulates the tumor suppressor gene Programmed cell death 4 (PDCD4), thereby promoting the development of gastric cancer [16]. It is important to note that compared with precancerous lesions and normal tissues, ovarian cancer has low expression of miR-93, and the expression level of miR-93 is negatively correlated with the differentiation grade and FIGO stage of ovarian cancer [17].Therefore, it suggested that miR-93-5p is a tumor-related miRNA implicated in various of tumor type.

We demonstrated CCND2 could be a candidate target gene of miR-93-5p. CCND2 is a member of the cyclin family and is a key positive regulator of the mammalian cell cycle and always upregulated in carcinogenesis of tumors [9]. The complex formed by CCND2 and CDK4/6 (CCND2-CDK4/6) regulates the phosphorylation and inactivation of tumor suppressor retinoblastoma protein (RB) to prompt cells transition from G1 phase to S phase [10]. CCND2 upregulated and could be a therapeutic target for a variety of tumors, including colon cancer, stomach cancer, liver cancer, breast cancer and lung cancer, etc. [18–21]. We found that the expression of CCND2 in epithelial ovarian cancer tissue and epithelial ovarian cancer cell A2780 was higher than that of normal ovarian tissue and normal epithelial ovarian cancer cell IOSE80, respectively. The CCK8 assay showed that the cell proliferation ability decreased and the flow cytometry showed that the proportion of cells in the G1 phase of the cell cycle increased indicated miR-93-5p suppress cell cycle S phase transition which may answer the role of miR-93-5p inhibition of cell proliferation. Interestingly, transfection of miR-93-5p mimic into ovarian cancer cell can negatively regulate the expression of CCND2 mRNA and protein level. Luciferase reporter assay showed miR93-5p can partly reduce CCND2 by binding its 3’UTR. We found cell migration and apoptosis did not too much significantly suppressed by miR-93-5p which may result from miR-93-5p promoting degradation mRNA of CCND2. Finally, we confirmed that miR-93-5p and CCND2 expression is negatively correlated in tumor tissues of patients with ovarian cancer. These data indicated that upregulation of CCND2 by reduction of miR-93-5p probably is conducive to ovarian cancer tumor growth and survival. Our finding partly revealed the mechanism of miR-93-5p in the evolution of ovarian cancer cells and its clinical potential in the battle against ovarian cancer.

## Data Availability

The authors confirm that the data supporting the findings of this study are available within the article.
